# Robust Beamforming Based on Graph Attention Networks for IRS-Assisted Satellite IoT Communications

**DOI:** 10.3390/e24030326

**Published:** 2022-02-24

**Authors:** Hailin Cao, Wang Zhu, Wenjuan Feng, Jin Fan

**Affiliations:** 1 Chongqing Key Laboratory of Space Information Network and Intelligent Information Fusion, Chongqing University, Chongqing 400044, China; zhuwang1224@cqu.edu.cn (W.Z.); 20173581@cqu.edu.cn (W.F.); 2 National Astronomy Observatory of China, Chinese Academy of Sciences, Beijing 100012, China; jfan@bao.ac.cn

**Keywords:** intelligent reflecting surface, low Earth orbit satellite, graph attention networks, unsupervised learning, beamforming

## Abstract

Satellite communication is expected to play a vital role in realizing Internet of Remote Things (IoRT) applications. This article considers an intelligent reflecting surface (IRS)-assisted downlink low Earth orbit (LEO) satellite communication network, where IRS provides additional reflective links to enhance the intended signal power. We aim to maximize the sum-rate of all the terrestrial users by jointly optimizing the satellite’s precoding matrix and IRS’s phase shifts. However, it is difficult to directly acquire the instantaneous channel state information (CSI) and optimal phase shifts of IRS due to the high mobility of LEO and the passive nature of reflective elements. Moreover, most conventional solution algorithms suffer from high computational complexity and are not applicable to these dynamic scenarios. A robust beamforming design based on graph attention networks (RBF-GAT) is proposed to establish a direct mapping from the received pilots and dynamic network topology to the satellite and IRS’s beamforming, which is trained offline using the unsupervised learning approach. The simulation results corroborate that the proposed RBF-GAT approach can achieve more than 95% of the performance provided by the upper bound with low complexity.

## 1. Introduction

With the advantages of providing global-coverage, high-throughput capability, and low-cost internet access, satellite communication has drawn significant attention from both industry and academia and is regarded as a promising solution for meeting the needs of the Internet of Remote Things (IoRT) [1,2]. Currently, there are three types of satellites in space that provide global service, including geosynchronous Earth orbit (GEO) satellites, medium Earth orbit (MEO) satellites and low Earth orbit (LEO) satellites [3]. Compared with GEO and MEO satellites, LEO satellites have recently become popular due to their lower development costs, better signal strength, and the potential for large-scale LEO satellite networks that can guarantee lower transmission delays [4,5]. More than 40,000 LEO satellites are planned by SpaceX Starlink alone. On the other hand, LEO satellites are deployed at an altitude of 500–1500 km with an orbital period shorter than 2 h. The fast movement of LEO satellites results in a very limited window for transmission to ground devices, approximately 10 min/pass [6]. The maximum completion time optimization for the Internet of Things (IoT) in LEO satellite-terrestrial integrated networks (STINs) was investigated in [7], and a cooperative nonorthogonal multiple access (NOMA) scheme for data transmission was proposed. As the amount of data transferred continues to increase, it becomes increasingly challenging for LEO satellites to transmit all data within such a small transmission window, especially in remote rural areas with no terrestrial infrastructure. Moreover, in practical satellite systems, line-of-sight (LoS) communication between satellite and terrestrial users is difficult to maintain due to obstacles and shadowing [8]. In [9], the authors investigated unmanned aerial vehicle (UAV) swarms and LEO satellite constellation-assisted data collection for IoRT networks, where UAV swarms were used as relays to improve the channel environment. UAVs have the benefits of high mobility, flexible deployment, and LoS transmission [10,11]. However, when introducing UAVs in LEO-assisted IoRT networks, data transmission becomes more challenging because of the different channel characteristics of UAV-ground and UAV-satellite links, as well as the battery and cache capacity limitations of UAVs.

Considered promising technology, intelligent reflecting surfaces (IRSs) have recently received substantial attention [12,13,14,15,16]. IRS consists of a large number of passive elements which can introduce controllable phase shifts. Intelligently adjusting these phases can change the reflected signal propagation. Therefore, it has been widely deployed in wireless communication systems to enhance the intended signal power at the receiver or mitigate the cochannel interference [14,15,16]. Refs. [14,15] investigated the weighted sum rate and transmitted power in an IRS-aided MISO system, respectively, by jointly optimizing the transmit beamforming vectors at the base stations (BSs) and the reflective beamforming vector at the IRS. Ref. [16] studied the secure transmission optimization for IRS-assisted STINs.

Most of the existing literature assumes that the perfect channel state information (CSI) is known. However, this assumption is impractical because the number of IRS reflecting elements is large and not capable of performing active transmission/reception and signal processing [17]. Previous studies proposed various channel estimation schemes in the IRS-assisted multiuser system [17,18,19,20]. However, the abovementioned methods are based on the BSs have fixed locations, and the variation of BS-IRS common channel is feeble. These approaches cannot be efficiently applied to the IRS-assisted LEO satellite system since the high-speed scenarios with fast time-varying channels would be updated more frequently. In actual deployment, the high complexity channel estimation schemes and beamforming algorithms may cause the instantaneous CSI obtained by LEO satellites to be out of date [21], which would dramatically diminish the system’s performance.

Fortunately, artificial intelligence (AI) technology provides simple approaches to address such complex problems [21,22,23,24]. Yang et al. [22] investigated secure physical communication based on IRS under the condition of time-varying channel coefficients and proposed a deep reinforcement learning approach to jointly optimize both BS and IRS beamforming. Ge et al. [23] established a deep transfer learning framework to solve the beamforming optimization problem for the IRS-assisted MISO system. Jiang et al. [24] trained a graph neural network (GNN) architecture to directly map the received pilots to the IRS’s phase shifts and BS beamforming matrix. However, Ref. [24] merges user location and pilots directly, resulting in the location features being easily ignored. In addition, due to the long distance between the satellite and users, the pilots received by the satellite is insensitive to user position information. Therefore, this approach cannot be effectively utilized in our system. Note that existing research on IRS mainly focuses on the ground cellular network system, and most research has been based on a static environment. When the application is extended to LEO satellite and the users’ location may also change, the algorithm’s computational complexity has a significant impact on performance.

In this paper, we commit to applying AI technology to solve the complex beamforming design problem in an IRS-assisted LEO satellite communication system. Specifically, the IRS is used to provide additional reflective links to overcome the serious attenuation caused by occlusion between the LEO satellites and users’ direct links. In our work, we establish a global optimization problem for maximizing the sum-rate of mobile users by optimizing both active and passive beamforming schemes jointly. To solve this problem, we propose a robust beamforming approach based on graph attention networks (RBF-GAT). Here are the main contributions of this paper:

First, we propose a novel architecture for LEO satellite IoT networks assisted with IRS. A deep neural network (DNN) architecture based on graph attention networks (GAT) [25] is constructed to capture dynamic network topology in real-time as a result of the mobility of the satellite and its users.

Second, a composite neural network combining a GAT layer and multiple fully connected (FC) layers is used to directly map the received pilots and network topology to the satellite and IRS beamforming, eliminating the necessity for channel estimation. To reduce the complexity of the RBF-GAT model, the mapping neural networks of satellite and IRS use the same feature extraction layers before the last normalization layer.

Third, we define a loss function to implement unsupervised training offline, thereby avoiding the labeling overhead that occurs in traditional supervised learning. Simulation results demonstrate that the proposed RBF-GAT approach, in well-trained conditions, will be able to approach upper-bound sum rate with a low level of computational complexity.

The remainder of this paper is organized as follows. Section 2 introduces the IRS-assisted LEO satellite communication system model. Section 3 describes the detailed architecture of RBF-GAT and the training process. Section 4 gives the simulation results and the complexity analysis. Section 5 presents the concluding remarks of this paper.

Notations: Boldface letters are used to denote vectors or matrices. ${\mathbb{C}}^{m\times n}$, ${\mathbb{R}}^{m\times n}$, and ${\mathbb{R}}^{m}$ represent the m×n complex, real matrices and m-dimensional real vector, respectively. The distribution of complex Gaussian random variables with mean μ and variance $\sigma_{}^{2}$ are denoted by $\left(\mu_{},\sigma_{}^{2}\right)$. The term diag(⋅) denotes the diagonalization of the vector, and ${\left(\cdot \right)}^{T}$ denotes the transpose of the matrices. The symbol ∘ denotes the Hadamard product.

## 2. System Model

### 2.1. Signal Model

**Figure 1:**
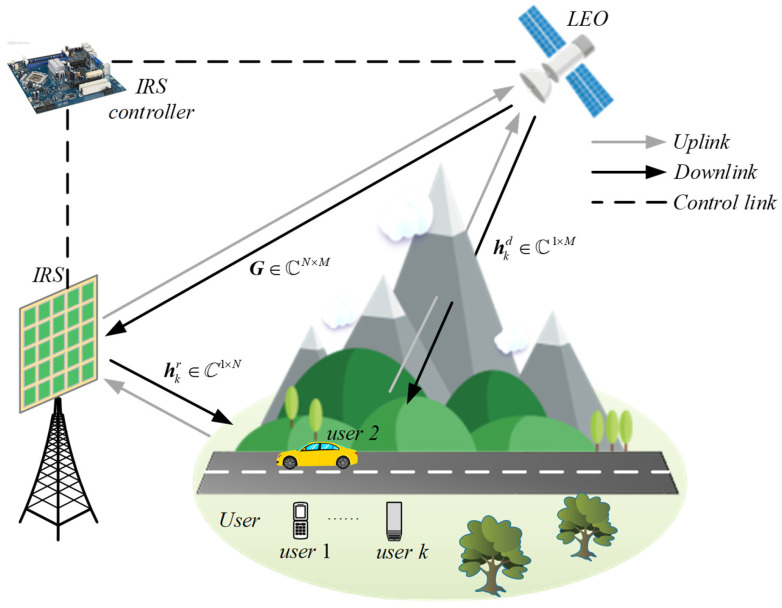
IRS-assisted downlink LEO satellite communication system.

As illustrated in Figure 1, this paper considers an IRS-assisted downlink LEO satellite communication system in IoRT networks. The LEO satellite is equipped with an array-fed reflector antenna, which comprises *M* feeds and can mostly provide *M* beams. Within the coverage area of a satellite beam, there are *K* randomly distributed single-antenna mobile users. The direct link between the satellite and users suffers severe attenuation due to heavy shadowing, thus, an IRS is implemented to assist the communications. The IRS is composed of $N=N_{t}\times N_{t}$ reflecting elements and is attached to a smart controller for tuning phase shifts at each reflecting element. $N_{t}$ represents the number of array elements uniformly placed along the axis. The channels from the LEO satellite to IRS and user *k*,k=1,⋯,K are denoted by $G\in {\mathbb{C}}^{N\times M}$ and $h_{k}^{d}\in {\mathbb{C}}^{\;1\times M}$, respectively, while that from the IRS to user *k* is denoted by $h_{k}^{r}\in {\mathbb{C}}^{\;1\times N}$. As such, we let $\Theta \;=\;\mathrm{diag}[e^{j\theta_{1}},\cdots ,e^{j\theta_{N}}]$ be the phase shift matrix of IRS, where $\theta_{n}\in [0,2\pi ]\;$ is the phase shift of the *n*-th reflection element.

This paper considers that the LEO satellite carries out superposition coding before broadcasting signals to the users. Thus, the transmitted signal for all users at the satellite at time *t* is written as $x(t)=\sum_{k=1}^{K}w_{k}x_{k}(t)$, where $w_{k}\in {\mathbb{C}}^{\;M\times 1}$ and $x_{k}(t)$ represent the precoding vector and transmitted symbol for the *k*-th user, respectively. Hence, the signal received by the *k*-th user can be given as [15]:

(1) $$y_{k}(t)=\left(h_{k}^{d}+h_{k}^{r}\Theta G\right)\sum_{i=1}^{K}w_{i}x_{i}(t)+n_{k}(t)$$

where $n_{k}(t)$ is additive white Gaussian noise (AWGN) at the *k*-th user with a zero mean and unit variance. Accordingly, the signal-to-interference plus noise ratio (SINR) of user *k* can be expressed as:

(2) $$SNIR_{k}=\frac{{\left|\left(h_{k}^{d}+h_{k}^{r}\Theta G\right)w_{k}\right|}^{2}}{{\sum_{i\neq k}\left|\left(h_{k}^{d}+h_{k}^{r}\Theta G\right)w_{i}\right|}^{2}+\sigma_{k}^{2}}$$

In addition, we assume that the Doppler shift caused by the LEO satellite and users’ mobility can be perfectly compensated at the received end. Therefore, we ignore its influence in the following.

### 2.2. Channel Model

To realistically model the propagation characteristics of the satellite channel, the impact of path loss, atmospheric attenuation, and satellite beam gain should be accounted for. The downlink channel between the satellite and the ground device *k* can be expressed as [26]:

(3) $$h_{k}^{}=\sqrt{C_{k}}b_{k}^{\frac{1}{2}}\circ r_{k}^{\frac{1}{2}}\circ {\tilde{h}}_{k},\;k=0,1,\cdots ,K$$

where $C_{k}$ is the large-scale fading efficient, which can be calculated by:

(4) $$C_{k}={\left(\frac{\lambda }{4\pi d_{k}}\right)}^{2}\frac{G_{k}}{\kappa TB}$$

where $d_{k}$, λ and $G_{k}$ represent the propagation distance, carrier wavelength and receive antenna gain, respectively. $\kappa =1.38\times 10^{-23}J/m$ is Boltzman’s constant, B is the carrier bandwidth, and T represents the receive noise temperature. $b_{k}=[b_{k,1},\cdots ,b_{k,M}{]}^{T}$ in Equation (3) is an *M*-dimensional beam radiation pattern vector, where the *m*-th element $b_{k}$ can be approximated by [26,27]:

(5) $$b_{k,m}=b_{\max}{\left(\frac{J_{1}\left(u_{k,m}\right)}{2u_{k,m}}+36\frac{J_{3}\left(u_{k,m}\right)}{2u_{k,m}^{2}}\right)}^{2}$$

where $u_{k,m}=2.07123\sin(\varphi_{k,m})/\sin(\varphi_{k,3dB})$, $\varphi_{k,m}$ is the angle between the *m*-th satellite beam centre and user *k*, $\varphi_{k,3dB}$ is the 3-dB angle for the *k*-th user. $b_{\max}$ is the maximal satellite antenna gain. $J_{1}\left(\cdot \right)$ and $J_{3}\left(\cdot \right)$ are the first and third orders of the first-kind Bessel function, respectively. Moreover, $r_{k}$ in Equation (3) is also an *M*-dimensional vector in which represents the rain attenuation coefficient and its form of dB follows lognormal random distribution $\ln\left(20\mathrm{lg}(r_{k,m})\right)\sim (\mu_{m},\sigma_{m}^{2})$. Moreover, we adopt shadowed Rician fading as the satellite channel fading model, which is proposed in [28] and has been widely used in prior studies. In this model, the probability density function of $|{\tilde{h}}_{k}{|}^{2}$ can be expressed as:

(6) f|h˜k|2(x)=(2λkmk2λkmk+Ωk)mk12λkexp(−x2λk)⋅F11(mk,1,Ωkx2λk(2λkmk+Ωk)),x≥0

where F11(a;b;c) is the confluent hypergeometric function and $2\lambda_{k}$ and $\Omega_{k}$ are the average power of the scatter component and LoS component, respectively. $m_{k}\geq 0$ denotes the Nakagami-*m* parameter. Therefore, the channel fading coefficient ${\tilde{h}}_{k}$ can be represented as ${\tilde{h}}_{k}=(\lambda_{k},m_{k},\Omega_{k})$. When *k* = 0, we denote $h_{0}^{}$ represents the channel between the satellite and IRS. In this paper, we assume that the channels of satellite-IRS links undergo infrequent light shadowing (ILS), while satellite-user links experience frequent heavy shadowing (FHS) [29], i.e., $h_{0}^{}>h_{k}^{},\forall k\in \{1,2,\cdots ,K\}$.

For the channel model between IRS and user *k*, both LoS and no line-of-sight (NLoS) components are considered, so we model channel $h_{k}^{r}$ as Rician fading channels:

(7) $$h_{k}^{r}=\beta_{k}\left(\sqrt{\frac{\xi }{1+\xi }}{\tilde{h}}_{k}^{los}+\sqrt{\frac{1}{1+\xi }}{\tilde{h}}_{k}^{nlos}\right)$$

where $\beta_{k}$ is the path loss from the IRS to user *k* and can be modelled as $30+22\log(d_{k})$, $d_{k}$ is the distance between the RIS and the *k*-th user. ξ is the Rician factor, and ${\tilde{h}}_{k}^{nlos}$ is the NLoS component vector, which is a complex Gaussian distributed with zero mean and unit variance. Moreover, ${\tilde{h}}_{k}^{los}={\left[a{\left(\phi_{k},\psi_{k}\right)}_{1},\cdots ,a{\left(\phi_{k},\psi_{k}\right)}_{N}\right]}^{T}$ represents the LoS component vector, and the *n*-th element $a{\left(\phi_{k},\psi_{k}\right)}_{n}$ can be given by:

(8) $$a{\left(\phi_{k},\psi_{k}\right)}_{n}=e^{j\frac{2\pi d_{}}{\lambda }\left\{s_{n}\sin(\phi_{k})\cos(\psi_{k})+i_{n}\sin(\psi_{k})\right\}}$$

where $s_{n}=\mathrm{mod}(n-1,N_{t})$ and $i_{n}=\left\lfloor (n-1)/N_{t}\right\rfloor$. $\phi_{k}$ and $\psi_{k}$ are the azimuth and elevation angles of arrival (AoA) from the IRS to user *k*. *d* is the interelement spacing of IRS, and we assume d/λ=2.

### 2.3. Problem Formulation

This paper aims to enable LEO satellites to transmit as much data as possible within a limited time window. Thus, we investigate a sum-rate maximization problem by jointly optimizing the precoding matrix at the LEO satellite and reflect beamforming at the IRS, which can be given as:

(9) $$P1:\;\underset{w,\Theta }{\max}\;\;\sum_{k=1}^{K}R_{k}\;s.t.\;\;\;\theta_{n}\in [0,2\pi ],\;\forall n\in \{1,2,\cdots ,N\},\;\sum_{k=1}^{K}{\left\|w_{k}\right\|}^{2}\leq P_{L}.$$

where the constraints of (9) are the phase shift of IRS and the maximum transmit power of the LEO satellite, respectively. Due to the objective function (9) is nonconvex [14], and the traditional optimization algorithms usually require many iterations and are not suitable for high-speed scenarios. To solve the problem with low complexity, we propose an RBF-GAT to establish a direct mapping from the received pilots and network topology to the satellite and IRS beamforming.

## 3. Proposed RBF-GAT Framework

To acquire the downlink instantaneous CSI of the LEO satellite, we follow the literature [19] and propose a pilot transmission strategy to design the uplink pilots and the IRS phase shifts in the pilot phase. Specifically, all users send their pilot sequences with length *L* to the satellite simultaneously. Each pilot can be decorrelated at the LEO satellite because all users’ pilot sequences are designed to be orthogonal. We denote the received pilots of user *k* at the satellite as $p_{k}$, which contains rich CSI between satellite and user *k*. The conventional approach of acquiring CSI uses minimum mean-squared error (MMSE), and its calculation is very complicated, especially for the IRS cascade channel.

Notably, GAT is an effective way to process structured data that are represented as a graph. In this work, the distribution of users can be regarded as a graph. *K* users constitute nodes of the graph, and each node is encoded as a feature vector denoted as $a_{k}$, which is transmitted to the satellite via uplink and contains the common features (e.g., the locations of IRS and satellite) and the private features (e.g., user locations, category and priority). GAT can track the spatial fluctuations of the network in real-time by processing this feature.

In this section, we commit to training an RBF-GAT network to directly establish the mapping from $p_{k}$ and $a_{k}$ to the precoding matrix and reflect beamforming to maximize the system sum rate. We first introduce the RBF-GAT architecture in detail and then discuss the unsupervised training approach.

### 3.1. RBF-GAT Architecture

**Figure 2:**
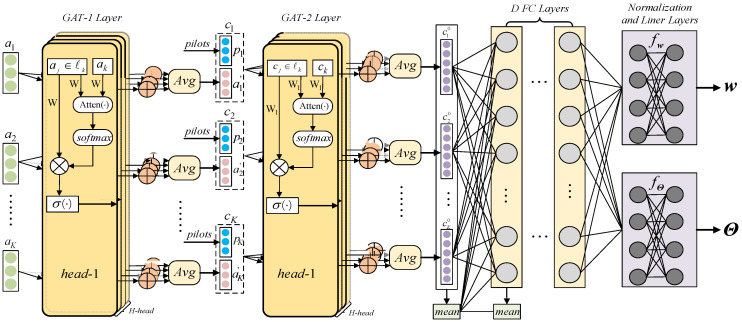
The architecture of the proposed RBF-GAT.

Our network consists of multiple GATs layers and multiple FC layers, as illustrated in Figure 2. First, for the raw feature $a_{k}$ obtained from the scenario, we need to map such vectors into a higher-dimensional space by a GAT layer, since the raw low-dimension feature contains less network topological information. The input to the GAT layer is a set of node features, $a=\{a_{1},a_{2},\cdots ,a_{K}\},a_{k}\in {\mathbb{R}}^{F}$, where F is the dimension of raw features in each user. To transform $a_{k}$ into a higher-level feature space of $F^{\prime }$ dimension, a shared weight matrix, $W\in {\mathbb{R}}^{F^{\prime }\times F}$, is applied to perform a linear transformation. We implement a shared self-attention mechanism Atten(⋅,⋅) to calculate the attention coefficients of the user and its adjacent users:

(10) $$e_{ij}=\mathrm{Atten}(Wa_{i},Wa_{j}),\;\mathrm{Atten}:{\mathbb{R}}^{F}\times {\mathbb{R}}^{F^{\prime }}\to \mathbb{R}$$

Note that to indicate the topological information of the network, we computed the attention coefficients only when the distance between user *i* and user *j* was within certain threshold. For easy comparison, a softmax function is applied to normalize the coefficients across different adjacent users, and the final normalized coefficients $\alpha_{ij}$ are obtained as:

(11) $$\alpha_{ij}=\mathrm{softmax}(e_{ij})=\frac{\exp(e_{ij})}{\sum_{j\in l_{i}}\exp(e_{ij})}$$

where $l_{i}$ is the set of adjacent users including itself in the current neighbourhood scope of the *i*-th user. Then, these coefficients are used to calculate a linear combination of the features to produce the output features for the current network user:

(12) $$a_{k}^{\prime }=\sigma \left(\sum_{j\in l_{k}}\alpha_{kj}Wa_{j}\right)$$

where σ(⋅) represents a nonlinear activation function and $a_{k}^{\prime }\in {\mathbb{R}}^{F^{\prime }}$ is the output vector of the single-head attention mechanism. To make the self-attention learning process more stable, a multi-head attention mechanism is used in this paper, which can be regarded as multiple single-head attentions executed independently in parallel, and taking the average as the output, can be represented by:

(13) $$a_{k}^{\prime }=\sigma \left(\frac{1}{H}\sum_{h=1}^{H}\sum_{j\in l_{k}}\alpha_{{}_{kj}}^{h}W^{h}a_{j}\right)$$

where *H* is the number of attention heads, $W^{h}$ represents the shared weight matrix of the *h*-th attention head.

Then, we concatenate the received pilot $p_{k}$ and the output features $a_{k}^{\prime }$ as the composite features of user *k* and denote them as $c_{k}$, which is a $\left(2ML+F^{\prime }\right)$ dimensional vector because all received pilots are decomposed into real and imaginary parts.

(14) $$c_{k}=[(p_{k}^{}{)}^{T},\;{\left(a_{k}^{\prime }\right)}^{T}{]}^{T}$$

Significantly, $c_{k}$ contains rich information about both the instantaneous CSI and network topology structure, so we use it as the input to the composite neural network. After $c_{k}$ pass through the GAT-2 layer, *D* FC layers and a normalization layer, the final output can be mapped directly to the precoding matrix of the satellite and the phase shift of the IRS.

We denote $c_{k}^{0}$ as the output of the second GAT layer, which is also the input of the first FC layer. According to Equation (13), $c_{k}^{0}$ can be expressed as:

(15) $$c_{k}^{0}=\sigma \left(\frac{1}{H}\sum_{h=1}^{H}\sum_{j\in l_{k}}\alpha_{kj}^{\prime h}W_{1}{}^{h}c_{k}\right)$$

where $\alpha_{kj}^{\prime h}$ and $W_{1}{}^{h}\in {\mathbb{R}}^{F^{\prime \prime }\times (2ML+F^{\prime })}$ are the normalized attention coefficients and the shared weight matrix of the *h*-th multihead attentions, respectively. In addition, we chosethe node-wise mean function as an aggregation function to aggregate the output characteristics of the GAT layer and concatenate it into each FC layer by using skip connect.

After *D* FC layers, the final output vectors, denoted as $c_{k}^{D}$, are passed to the normalization layer to produce the precoding matrix w and phase shift matrix Θ while ensuring the constraint of phase shift and transmit power. As with [20], we input $c_{k}^{D}$ to the linear layer $f_{w}(\cdot )$ with 2*MK* FC units and the linear layer $f_{\Theta }(\cdot )$ with 2*N* FC units. Then, the normalization layer outputs the real and imaginary components of the optimization variables. Finally, the complex solution can be obtained by combining the real and imaginary components.

### 3.2. Unsupervised Training

Since it is difficult for the IRS-assisted LEO satellite system to obtain data labels, we cannot train it by a classic deep learning algorithm with supervised learning techniques. Thus, unsupervised training is adopted for training the network. We define the loss function as:

(16) $$Loss=-\frac{1}{T}\sum_{i=1}^{T}\sum_{k=1}^{K}\omega_{k}R_{k}$$

where T represents the total number of training samples in a batch. To generate a training dataset, first, we generate the channel data according to the channel model discussed in Section 2. Then, all users transmit orthogonal pilot signals and additional information (i.e., location, priority, etc.) to the satellite. The LEO satellite can recover the pilots of all users from the received pilots and use it as part of the input of the neural network. The details of the training procedure are summarized in Algorithm 1.

Note that our model is trained offline, thus, the training process does not increase its computational complexity. During training, we use the stochastic gradient descent method to update the neural network parameters to minimize the loss function, which is equivalent to maximizing the objective function P1.
**Algorithm 1** Training procedure for RBF-GAT.**Input:** learning rate α, maximal epoch $E_{p}$, batch size $N_{b}$, training samples T,$I_{ite}$.**Output:** Optimal network weights parameter Φ1: Randomly initialize network parameter Φ.2: Calculate loss $L_{0}$ according to Equation (16) and initialize s=0.3: **for** $i_{}=1,\cdots ,E_{p}$ **do**4:        **for**
$j_{ite}=1,\cdots ,I_{ite}$ **do**
5:               Initialize the phase shift matrix Θ and generate received pilot according to [19] 6:               Randomly select T samples to compose a batch task.7:               Update the network weights parameter as $\Phi^{\prime }$ by Adam optimizer.8:        **end for**9:        Calculate loss $L_{i}$ according to Equation (16).10:        **if** $L_{i}>L_{i-1}$ **do**11:               Set s=0, $\Phi \leftarrow \Phi^{\prime }$ and save network weights.12:        **else**
13:               Update s←s+1 and judge whether the learning rate α needs to be updated.14:        **end if**15: **end for**

## 4. Simulation and Numerical Results

This section uses numerical simulations to evaluate the performance achieved by the proposed RBF-GAT for the sum-rate maximization problem. We first set the simulation parameters of the training neural network and IRS-assisted LEO satellite communication system. Then, we compare the RBF-GAT with several benchmarks proposed in prior works. Finally, we show the simulation results and analyze the computational complexities of the proposed RBF-GAT method. The simulation experiments conducted in this study were performed on a computer equipped with an Intel(R) Core(TM) i7-8700 processor @3.19 GHz, 64 GB RAM. The simulation platform utilized Python 3.6, and the neural network in the RBF-GAT was constructed using TensorFlow 1.6.

### 4.1. Simulation Parameter Setting

We use four attention heads (*H* = 4) and adopt three FCs (*D* = 3) in the proposed network. The names of the FC layers are denoted as $f_{1}$,$f_{2}$ and $f_{3}$, respectively. In addition, we set $a_{k}$ as a 10-dimensional vector (*F* = 10) that contains the priority information and the location information of user k, IRS and LEO satellites. The parameters of all layers are summarized in Table 1.

For network training, we use the Adam optimizer with an initial learning rate α=0.001, the number of maximal epochs $E_{p}$ is set to 350, and for each epoch, we generate $I_{ite}=100$ iterations to update the weights of the network. The batch size $N_{b}$ is set to 1000. To accelerate the convergence, the learning rate decays by a factor of 0.3 when the validation loss does not decrease for 5 consecutive epochs. Due to the statistical properties of the channel and the noise in the uplink pilot transmission, all the calculation results are generated based on averaging over 1000 instances.

For the considered IRS-assisted LEO satellite system, we assume that the LEO satellite altitude is 1000 km and that the satellite is equipped with *M* = 8 antennas. An IRS with 64 passive element locations at (0,0) and height 20 m. IRS is configured as an 8 × 8 uniform rectangular array. There are 6 terrestrial users uniformly distributed in a square area of [0, 200] m × [0, 200] m. We set the length of the uplink pilots to *L* = 20 for each user, and the user’s transmission power to 15 dBm. The uplink channels from users to IRS, from IRS to the LEO satellite, and from users to the LEO satellite are generated according to the channel model discussed in Section 2. The details of the coefficients are given in Table 2.

### 4.2. Benchmark Schemes for Comparison

After the RBF-GAT was trained offline, we compared its performance with the following benchmarks:

Upper Bound: Let the CSI of all channels is perfectly known at the IRS and the LEO satellite, and we optimize the sum-rate maximization problem by the block coordinate descent (BCD) algorithm proposed in [14], which can be treated as the system performance upper bound but, in reality, it is difficult to realize. We stop the BCD algorithm after 2000 iterations.

Deep Learning(GNN): Adopt a GNN architecture proposed in [24] to capture the interactions among all users and the LEO satellite. The user locations and received pilots are directly concatenated as the input feature, and then train the model offline in an unsupervised manner.

Deep Learning(MLP): Design a multi-layer perceptron (MLP), which is composed of a simple network including multiple layers with several neurons, to establish the mapping from pilots and location to beamforming. This method has been studied in [30].

Without IRS: Let *N* = 0, and then the precoding matrix of the LEO satellite is optimized using the alternating optimization algorithm presented in [14].

Random Phase: The IRS phase shift matrix is initialized with random value, and the alternating optimization algorithm proposed in [14] is then applied to optimize the precoding matrix of the LEO satellite.

### 4.3. Numerical Results

In this subsection, we present the numerical results of the proposed approach. We assume that the users’ locations are fixed within a time slot, the time slot is small enough and the low-complexity RBF-GAT can implement active and passive beamforming within the time slot.

**Figure 3:**
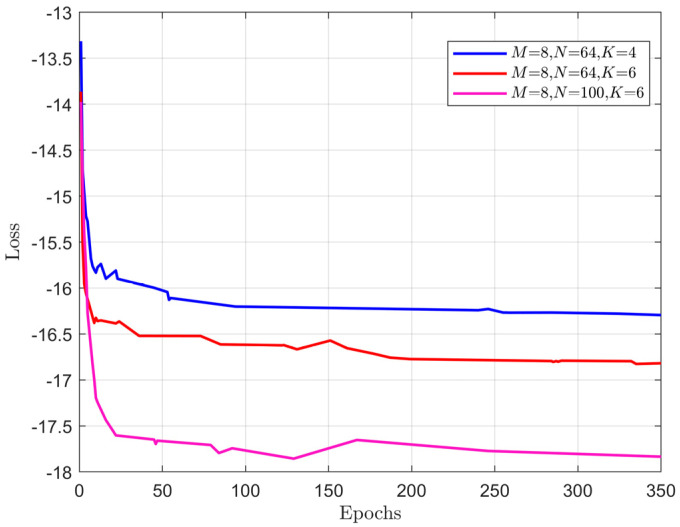
The convergence rate with different training parameters.

To verify the convergence rate of the proposed RBF-GAT scheme, we plot the loss value during training versus the number of epochs with three different training parameters. Figure 3 shows that the proposed scheme converges to a locally optimal solution in less than 200 training epochs. In addition, the smaller the number of IRS reflection elements or users, the faster the convergence speed of the algorithm. This is because the number of users or IRS elements is positively correlated with the number of weight parameters to be trained.

**Figure 4:**
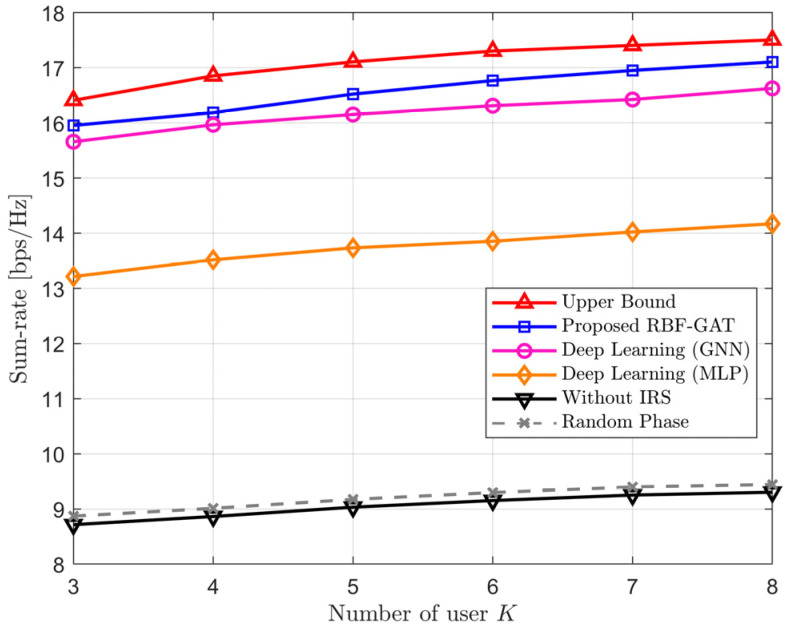
The sum-rate versus the number of users *K* with *M* = 8, *N* = 64, *L* = 20 and $P_{L}=30\;\mathrm{dBW}$.

Figure 4 shows the sum-rate versus the number of users under different schemes. The sum-rates under all considered schemes increase with an increasing number of users *K*, and the larger the value of *K* is, the slower the growth trend. This phenomenon can be explained by the concavity of the log function from the sum rate. It is seen that those methods aided with IRS observably exceed the one without IRS, and the performance gain by deploying IRS is inappreciable if the phase shift matrix is initialized by random value. Moreover, both RBF-GAT and GNN can achieve performance close to the upper boundary, but the proposed RBF-GAT consistently outperforms GNN, and the gap increases with *K*. The reason for the increase is that the LEO satellite is insensitive to user’s location information, in contrast to GNN directly merging the position coordinates of the user, RBF-GAT can effectively capture the dynamic network topology by GAT layers. Thus, the proposed RBF-GAT is more suitable for IRS-aided LEO satellite dynamic scenario communication.

**Figure 5:**
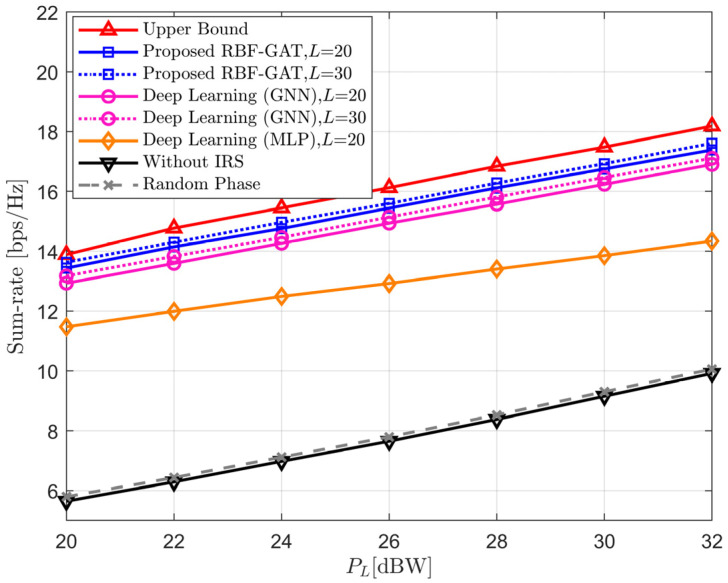
The sum-rate versus the LEO satellite transmit power *P_L_*.

Figure 5 illustrates the sum-rate of different schemes with respect to the transmit power $P_{L}$ when *N* = 64. It is observed that the sum-rate increases for all considered schemes as the transmission power increases, and the random phase method still has only a weak gain. As we expected, the performance of RBF-GAT is always closest to the upper boundary under the condition of equal pilot length. In addition, as the pilot length increases, the rate sum also increases but never exceeds the upper boundary. This is because the longer the pilot signal received is, the richer the CSI contained. Thus, more features can be learned by the neural network. On the other hand, increasing the pilot length will also lead to a larger delay in data transmission. In practical applications, we should make trade-offs according to different requirements.

**Figure 6:**
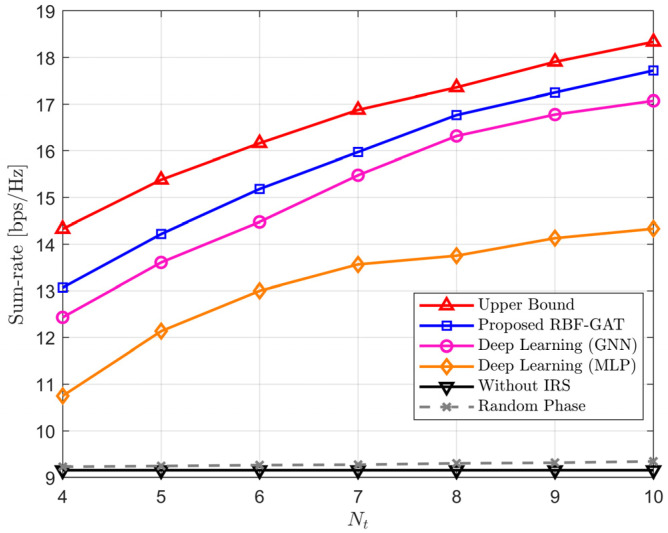
The sum-rate versus the number of IRS elements *N* = *N_t_* × *N_t_*.

Figure 6 shows the sum-rate versus the number of elements of the IRS in four different schemes. In Figure 6, we set the number of feeds at LEO satellite as *M* = 8, pilot length as *L* = 20, and the transmit power of LEO satellite as $P_{L}=30\;\mathrm{dBW}.$ We can find that the sum-rate of the without IRS method remains constant and the random phase method increase slightly as the numbers of IRS element grows. From Figure 5 and Figure 6, we can calculate that the proposed RBF-GAT approach can achieve more than 95% of the performance provided by the upper bound. In addition, both the number of IRS elements and the increase in transmit power can improve the sum-rate. However, compared to increasing the transmit power, increasing the IRS elements to improve the sum-rate performance is a more energy-efficient scheme due to the IRS elements being passive. The above numerical simulations further validate the robustness and effectiveness of our proposed RBF-GAT schemes.

### 4.4. Computational Complexity Analysis

The complexity of the BCD is $O(I_{o}(2NMK+KM^{2}+K^{2}M^{2}))$ [14], where $I_{o}$ is the number of iterations and does not include the complexity of the channel estimation. For IRSs with passive elements, the conventional least square channel estimation methods have a computational complexity of O(LKMN) with *L* < *NM*. In our proposed RBF-GAT method, the channel estimation is omitted. For the training stage, let *Z*_1_, *Z*_2_, and *Z*_3_ denote the number of neurons in the three FC layers in turn. The computational complexity of the proposed RBF-GAT scheme in each iteration is $O(KFF^{\prime }+KF^{\prime \prime }(2ML+F^{\prime })+F^{\prime \prime }Z_{1}+Z_{1}Z_{2}+Z_{2}Z_{3}+2Z_{3}MK+2Z_{3}N)$. In the training phase, the model is trained for $E_{p}$ epochs, with each epoch being $I_{ite}$ iterations. Hence, the total computational complexity of the proposed method is $O(E_{p}I_{ite}(KFF^{\prime }+KF^{\prime \prime }(2ML+F^{\prime })+F^{\prime \prime }Z_{1}+Z_{1}Z_{2}+Z_{2}Z_{3}+2Z_{3}MK+2Z_{3}N)).$ The high computational complexity training process is performed offline. Therefore, the actual computational complexity of our proposed method is only linear in *M*, *N* and *K*.

Due to the GNN and MLP methods are also established by neural networks, and they have approximate computational complexity as the proposed RBF-GAT. However, the proposed method achieves better performance, which is shown in the previous subsection. It is easy to see that the proposed RBF-GAT method has lower computational complexity and has significant advantages in the dynamic scenarios of satellite communication.

## 5. Conclusions

In this paper, we investigated the IRS-aided LEO satellite communication system. Specifically, we formulated a sum-rate maximization problem by optimizing the satellite precoding and IRS beamforming jointly. To tackle the time-varying network topology and high transmission delay of satellite communication, an RBF-GAT was presented to establish a direct mapping from the received pilots and network topology to the satellite and IRS beamforming, and the unsupervised learning mechanism was used to train this network offline. Compared with traditional beamforming methods, the proposed approach has the ability to capture the dynamic network topology and lower computational complexity. Therefore, it is more suitable for dynamic LEO satellite communication scenarios. The simulation results corroborated that the proposed scheme can achieve approximate performance compared with an optimal solution.

## Figures and Tables

**Table 1:** The parameters of RBF-GAT.

Layer Name	Input Dimension	Output Dimension	Activation Function
GAT1	*K* × 10	*K* × 512	LeakyReLU
GAT2	*K* × (512 + 2*ML*)	*K* × 1024	LeakyReLU
*f*_1_	*K* × 1024	2048	ReLU
*f*_2_	2048	1024	ReLU
*f*_3_	1024	512	ReLU
*f_w_*	512	2*MK*	ReLU
*f_Θ_*	512	2*N*	ReLU

**Table 2:** Simulation parameters in the IRS-assisted LEO satellite communication network.

Parameter	Definition	Value
$P_{L}$	Satellite maximum transmit power	30 dBW
ν/λ	Carrier frequency	20 GHz
$G_{k}/T$	User received gain per to noise temperature	15 dB/K
B	Bandwidth	25 MHz
$\varphi_{k,m}$	Beam angles between IRS/user and satellite	0.01°~0.5°
$\varphi_{k,3dB}$	3-dB angle	0.4°
$b_{max}$	Maximal satellite antenna gain.	52 dBi
$\mu_{m}$	Rain fading mean	−2.6 dB
$\sigma_{m}^{2}$	Rain fading variance	1.63 dB
ξ	Rician factor	10
${\tilde{h}}_{0}$	ILS fading parameters between satellite to IRS channel	(19.4, 0.158, 1.29)
${\tilde{h}}_{k}$	HFS fading parameters between satellite to user channel	$\left(0.739,\;0.063,\;8.97\times 10^{-4}\right)$

## Data Availability

Not applicable.
